# 2-Methyl-6-(phenylethynyl) pyridine (MPEP) reverses maze learning and PSD-95 deficits in *Fmr1* knock-out mice

**DOI:** 10.3389/fncel.2014.00070

**Published:** 2014-03-06

**Authors:** Réno M. Gandhi, Cary S. Kogan, Claude Messier

**Affiliations:** School of Psychology, University of OttawaOttawa, ON, Canada

**Keywords:** fragile X syndrome, Hebb-Williams mazes, 2-methyl-6-(phenylethynyl) pyridine, post-synaptic density-95, Western blot

## Abstract

Fragile X Syndrome (FXS) is caused by the lack of expression of the fragile X mental retardation protein (FMRP), which results in intellectual disability and other debilitating symptoms including impairment of visual-spatial functioning. FXS is the only single-gene disorder that is highly co-morbid with autism spectrum disorder and can therefore provide insight into its pathophysiology. Lack of FMRP results in altered group I metabotropic glutamate receptor (mGluR) signaling, which is a target for putative treatments. The Hebb-Williams (H-W) mazes are a set of increasingly complex spatial navigation problems that depend on intact hippocampal and thus mGluR-5 functioning. In the present investigation, we examined whether an antagonist of mGluR-5 would reverse previously described behavioral deficits in fragile X mental retardation 1 knock-out (*Fmr1* KO) mice. Mice were trained on a subset of the H-W mazes and then treated with either 20 mg/kg of an mGluR-5 antagonist, 2-Methyl-6-(phenylethynyl) pyridine (MPEP; *n* = 11) or an equivalent dose of saline (*n* = 11) prior to running test mazes. Latency and errors were dependent variables recorded during the test phase. Immediately after completing each test, marble-burying behavior was assessed, which confirmed that the drug treatment was pharmacologically active during maze learning. Although latency was not statistically different between the groups, MPEP treated *Fmr1* KO mice made significantly fewer errors on mazes deemed more difficult suggesting a reversal of the behavioral deficit. MPEP treated mice were also less perseverative and impulsive when navigating mazes. Furthermore, MPEP treatment reversed post-synaptic density-95 (PSD-95) protein deficits in *Fmr1* KO treated mice, whereas levels of a control protein (β-tubulin) remained unchanged. These data further validate MPEP as a potentially beneficial treatment for FXS. Our findings also suggest that adapted H-W mazes may be a useful tool to document alterations in behavioral functioning following pharmacological intervention in FXS.

## Introduction

Fragile X Syndrome (FXS) is a neurodevelopmental disorder that is caused by the loss of function mutation of the fragile X mental retardation 1 (*Fmr1*) gene on the X chromosome (reviewed in O’Donnell and Warren, 2002; Santoro et al., 2012; Online Mendelian Inheritance in Man ® [OMIM] 309550) resulting in lack of fragile X mental retardation protein (FMRP) expression (Fu et al., 1991; Pieretti et al., 1991). In turn, lack of FMRP results in a number of symptoms including disorders of intellectual development, attention deficit and hyperactivity, anxiety, epilepsy, as well as particular physical features such as an elongated face and macroorchidism (Hagerman, 1996; Turner et al., 1996; O’Donnell and Warren, 2002; Hatton et al., 2006; Sullivan et al., 2006; Scerif et al., 2007). Importantly, a large proportion of individuals (25–47%) affected by FXS display autistic behaviors or a co-morbid diagnosis of autism (Kaufmann et al., 2004; Hatton et al., 2006), making FXS the only clear genetically associated form of autism. Relevant to the present investigation, FXS patients display poorer performances as compared to developmentally matched participants on a number of different visual-spatial dependent tasks (Cornish et al., 1998, 1999; Kogan et al., 2004, 2009; MacLeod et al., 2010; Van der Molen et al., 2010).

In fragile X mental retardation 1 knock-out (*Fmr1* KO) mice an exaggerated form of mGluR mediated long-term depression (LTD) has been documented in hippocampal neurons (Huber et al., 2002) evidenced by elevated levels of “LTD” proteins at basal states (Nosyreva and Huber, 2006; Osterweil et al., 2010) and by the internalization of AMPA receptors (Snyder et al., 2001). Following the identification of, and much research on, LTD in *Fmr1* KO mice, the prevailing opinion is that Fmrp, which binds to approximately 4% of total brain mRNA (Brown et al., 2001; Darnell et al., 2011), acts as a translational suppressor of proteins *in vivo*, many of which are implicated in synaptic plasticity (Bassell and Warren, 2008; Darnell et al., 2011; Bhakar et al., 2012)**.**

It has been hypothesized that in the absence of the translational suppression functions of Fmrp, abnormally elongated spines develop and are responsible for some of the clinical manifestations of FXS such as disorders of intellectual development and audiogenic seizures (Bear et al., 2004; Krueger and Bear, 2011). Thus, intervention with antagonists that selectively target mGluR-5 has been promising in that these agents can mitigate signaling and as a result correct some of the downstream effects that occur in the absence of Fmrp. Consistent with group I mGluR-signaling as mediating prolonged LTD in *Fmr1* KO mice, one study employed small interfering RNA (siRNA) specific to the *Fmr1* gene sequence to demonstrate that reductions of Fmrp in dendrites of hippocampal neurons lead to an increase in the internalization of the AMPAR subunit, GluR1 (Nakamoto et al., 2007). Treatment with 2-methyl-6-phenylethynyl-pyridine (MPEP), an mGluR5-antagonist, rescued the abnormal AMPAR trafficking, an effect not found for NMDA receptors (NMDARs). In the absence of Fmrp and following 20 days of *in vitro* culturing, neurons from adult *Fmr1* KO mice were classified as having excess filopodia (spines with a long and thin appearance) relative to wild-type cultured neurons that had a mushroom shaped appearance with a large spine head (de Vrij et al., 2008). Treatment of *Fmr1* KO neurons with two different mGluR-5 antagonists (200 μM MPEP and 300 μM fenobam) for 4 h rescued the protrusion phenotype, restoring the spine/filopodia ratio in *Fmr1* KO neurons to the levels observed in wild-type neurons (de Vrij et al., 2008). Consistent with this finding, other researchers have reversed hippocampal spine elongations by using alternative mGluR-5 antagonists such as Mavoglurant (AFQ056; Levenga et al., 2011). Regarding cortical neurons, in one study, daily administration of 20 mg/kg of MPEP over the course of a week ameliorated average spine length and density in adult *Fmr1* KO mice without producing significant tolerance or toxicity effects (Su et al., 2011).

Arguably the strongest support for targeting mGluR- signaling with antagonists comes from research studies that cross-bred *Fmr1* KO mice with Grm5 mutant mice that have a 50% reduction of mGluR-5 expression (rather than a complete KO which would negatively impact brain function and lead to death). This procedure rescued several phenotypic aspects of the FXS mouse model. In this regard, reduction of mGluR-5 expression in *Fmr1* KO mice significantly reduced hippocampal LTD, rescued the increased density of long and thin spines, reduced the elevated basal protein synthesis rates and finally, reduced audiogenic seizures (Dölen et al., 2007).

Behaviorally, *Fmr1* KO mice of the hybrid strain C57Bl/6J X Friend Virus B NIH Jackson (FVB/NJ) displayed increased center square entries and duration during open field testing indicative of impulsivity and disinhibiton. Single intraperitoneal (i.p.) injection of either 10 or 30 mg/kg of MPEP rescued these deficits such that open field performance 30 min after injection was statistically indistinguishable from control mice (Yan et al., 2005).

Despite much progress with antagonism interventions, there remains a need for reliable and valid means of assessing improvement in patients receiving treatments, which are comparable to those used in animal studies. Typically human FXS studies attempting to assess progress in various cognitive domains have produced inconsistent findings as a result of outcome measures that are confounded by floor and ceiling effects (Berry-Kravis et al., 2006). We previously showed that Hebb-Williams (H-W) mazes are a viable visual-spatial assay for use with both FXS participants and KO mice. Both populations exhibit similar behavioral impairments (i.e., more errors than controls) (MacLeod et al., 2010). More recently, we demonstrated that Fmrp intact mice, but not *Fmr1* KO mice, evidenced upregulations of post-synaptic density-95 (PSD-95) following completion of the H-W mazes (Gandhi et al., 2014). Given that PSD-95 has been hypothesized as a key protein ostensibly involved in both AMPAR regulation and dendritic spine structure (Keith and El-Husseini, 2008), our data suggests that PSD-95 is a good candidate protein in order to examine the effects of antagonism treatment in *Fmr1* KO mice.

Thus, for the present study, we hypothesized that MPEP treatment of *Fmr1* KO mice would result in reversal of the previously described deficit (i.e., significantly fewer errors) on the H-W mazes as well as a reversal of the PSD-95 protein deficit relative to saline treated controls. We also report results from a manipulation check (i.e., a marble burying assay) experiment that confirms that the MPEP treatment remains active throughout maze testing. Specifically, when MPEP is pharmacologically active, marble burying (a repetitive behavior) is significantly reduced without a corresponding decline in locomotor activity (Thomas et al., 2012).

## Methods

### Animals

A total of 22, male, naïve *Fmr1* knock-out (KO) mice with a FVB background, bred from homozygote mating pairs that had been backcrossed for 11 generations, were obtained from Jackson Laboratories (FVB.129P2-*Fmr1*^tm1Cgr^/J; JAX Stock # 004624; Bar Harbor, ME, USA). These mice do not carry the rd1 mutation and consequently, do not develop retinal degeneration. The FVB genetic background was chosen in view of the documented modest visual-spatial abilities (Dobkin et al., 2000; Van Dam et al., 2000).

Mice were shipped at 4 weeks of age and were approximately 12 weeks old when they began experimental procedures. Mice were given 2 weeks to acclimate to the vivarium. During that time, they were housed in groups of four in standard (27 × 21 × 14 cm) polypropylene cages. All mice were kept on a 12 h light-dark cycle (light 07:00–19:00 h) in a temperature controlled environment (21°C) and fed Rodent Chow (Harlan Global, Mississauga, ON, Canada) and tap water. Eight days prior to testing, all mice were housed in individual cages. Behavioral testing took place during 08:00–15:00 h to reduce variability associated with diurnal rhythms. To ensure high levels of motivation during the study, mice were maintained at approximately 85–90% of their original body weight and were fed a food ration approximately 30 min after daily testing procedures ended. The ethics protocol was approved by the University of Ottawa Animal Care Committee (UOACC) and precautions were taken to minimize any pain or discomfort according to the guidelines of the Canadian Council on Animal Care (CCAC).

### Apparatus

The H-W test apparatus was constructed according to the specifications outlined by the developers, Rabinovitch and Rosvold (1951). Specifically, the maze was built using black opaque plexiglass and fitted with a translucent plexiglass cover top (Plastics of Ottawa Ltd., Ottawa, ON, Canada). The apparatus consisted of a large open area, square in shape (60 × 60 × 10 cm), with diagonally opposing start and goal box areas (20 × 10 × 10 cm). The start and goal box areas were equipped with sliding, removable plexiglass doors to control entry and confinement, covered by clear plexiglass lids. In the goal box, a recessed food cup (2.5 cm diameter) was placed in the center and baited with a 20 mg of Rodent Chow, during the latter phases of the experiment. The floor of the square open area was delineated by 36 equally sized squares. The squares were used as markers for manually placing barriers that defined different maze problems and error zones (Rabinovitch and Rosvold, 1951). The barriers (10 cm high) were constructed with black opaque plexiglass. Extra-maze cues were minimized by placing the apparatus on a desk table (100 × 75 cm) and by enclosing it within white wall coverings hanging from the ceiling.

### Drug treatment

Gq-coupled, group I metabotropic glutamate receptors (mGluR) consist of two different types of receptors. Treatments for FXS targeting mGluR-5 receptors have been favored over mGluR-1 receptors given that the latter produces motor deficits in animals (Berry-Kravis et al., 2011). As such, the mGluR-5 antagonist, MPEP, MW 229.70, (MPEP; Sigma Aldrich, Oakville, ON, Canada) was used in the current investigation. MPEP is a potent and selective antagonist of mGluR-5 that is able to cross the blood-brain barrier readily (Gasparini et al., 1999). Regarding preparation, drug powder was dissolved into a vehicle (saline) and aliquots containing 5mg/ml of stock solution were stored at −20°C. Thereafter single aliquots were allowed to warm to room temperature, briefly centrifuged, and MPEP treated mice received an intraperitoneal (i.p.) injection of 20 mg/kg based on their body weight on the day of testing. Similarly, aged matched control mice were administered an equivalent dose of saline without the drug based on their body weight. MPEP was previously reported to be biologically active from 15 to 75 min following i.p. injections (Yan et al., 2005). Based on this data and to allow sufficient time for the drug to take effect, mice in both groups were tested 30 min following drug or vehicle-only administration. The mg/kg dosage was determined based on a pilot study prior to experimentation (see data below).

### Pilot study—dose response determination

Studies using *Fmr1* KO mice and MPEP treatments (via an i.p. route of administration) followed by behavioral testing have attempted to optimize effective dose ranges from 0.05 mg/kg to 40 mg/kg (Yan et al., 2005; Su et al., 2011; Thomas et al., 2012). Based on the results from these studies, an initial pilot study was conducted with *Fmr1* KO mice (*N* = 6; Jackson Laboratories, Bar Harbor, ME, USA; FVB.129P2-*Fmr1*^tm1Cgr^/J; JAX Stock # 004624). Mice (*n* = 2) received vehicle, 20 mg/kg or 30 mg/kg of MPEP treatments over consecutive 4 days. Vehicle or MPEP treatments were administered twice per day (08:00 and 13:00 h) and mice were allowed 30 min to allow sufficient time for the drug to take effect prior to participating in the marble burying assay. The total number of marbles buried collapsed across 4 days and 8 marble burying trials was measured. Bonferroni adjusted independent sample *t*-tests (*α* = 0.05/3 = 0.017) indicated there was significantly less marbles buried by 20 mg/kg treated mice relative to vehicle treated mice (*t* = 9.40, *p* = 0.011); and by 30 mg/kg treated mice relative to controls (*t* = 12.80, *p* = 0.006). However, there were no differences in aggregated marbles buried between the two doses of MPEP treatment (*t* = 0.949, *p* = 0.433). As such, the dose used for MPEP treated mice in the current investigation was set at 20 mg/kg to avoid potential unwanted side effects from the higher dose.

### Marble burying

A marble burying assay (Thomas et al., 2009) was used to ensure that MPEP doses were biologically active before and after maze testing. This assay reflects repetitive digging behavior without habituation effects to burying even if marble presentations are repeated multiple times during the same day or across several days (Thomas et al., 2009). The number of marbles buried decreases following the administration of Grp I mGluR antagonists (Thomas et al., 2012) and MPEP treatment does not significantly reduce voluntary locomotor. Concerning the assay itself, 20 marbles of varying color were arranged (15 mm in diameter) in a 4 × 5 pattern on top of approximately three and a half cm of bedding (SANI-CHIP) using clean (27 × 21 × 14 cm) polypropylene cages. Approximately 4 cm of open space, clear of marbles was left at one end of each cage in order to place a single mouse into the apparatus. Each mouse was allotted 20 min to bury as many marbles as possible. Marbles were considered buried if they were covered by >50% of SANI-CHIP bedding.

### Procedure

All 22 *Fmr1* KO mice underwent behavioral testing with half of the animals (*n* = 11) receiving 20 mg/kg treatment of MPEP and the others an equivalent dose of vehicle only (*n* = 11). The experiment was conducted in three phases: habituation, acquisition and testing. During the habituation phase, the H-W apparatus was cleared of all barriers and each mouse was allowed 20 min/day on 4 consecutive days to explore the maze including the start and goal box areas. During the last 2 days, the goal box area was baited with 20 mg of Rodent Chow and each mouse had *ad lib* access to the food for the duration of the session.

**Figure 1:**
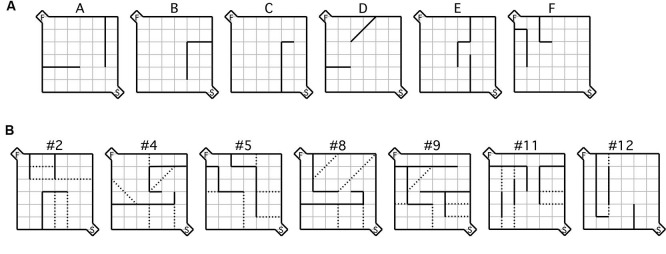
**Maze configurations. (A)** Testing was conducted during the acquisition phase using the six practice mazes labeled (A–F) and **(B)** the seven test mazes depicted, each of which was used during the testing phase. For each maze configuration, the (S) indicated in the bottom right hand corner represents the start box, while the (F) in the top left corner represents the goal box. Error zones are delineated by the dotted lines.

The acquisition phase consisted of training mice on six practice mazes (Figure 1A). Specifically, each mouse was trained for 2 sessions per day, the first starting at 08:00 h and the second at 13:00 h. Each session consisted of one of six possible practice mazes (five trials per maze) commencing with maze A. A trial was considered complete when the mouse entered the goal area and took a bite of food or 180 s had elapsed. Mice completed all six acquisition mazes in sequence (A–F) as many times as necessary for them to reach criterion; that of 2 consecutive sessions completed in less than 30 s each. The mean time to complete the acquisition phase was 11.6 days. Mice that were assigned to either MPEP or vehicle treatment during the subsequent phase (i.e., testing) did not differ in the number of days required to reach criterion in the acquisition phase (*t* = 0.18, *p* = 0.86).

Following acquisition, mice were given a selection of the standard test mazes (Figure 1B; Rabinovitch and Rosvold, 1951) based on the same procedures used during acquisition. 30 min prior to maze running mice were administered a either a dose of 20 mg/kg of MPEP or an equivalent volume of vehicle. Mice were then tested on a different maze in each session (five trials per maze) in the same order (i.e., #2, #4, #5, #8, #9, #11, #12) until all seven were completed, spanning 3.5 days/animal. The dependent measures of interest were latency and number of errors. Latency was recorded from the moment the barrier in the start box was raised until the animal took its first bite of food. An error was registered each time a mouse crossed its two front paws into a defined error zone (Figure 1B). Data from the testing phase were recorded using an overhead SONY camcorder and Media Cruise software (Thomson Canopus Co. Ltd., Kobe, Japan) on a standard desktop computer. Immediately after each maze, individual mice were placed in separate marble burying assays for 20 min each, following which the number of marbles buried was recorded. Over all phases of the study, the experimenter was never visible during the runs. To reduce odors from conspecifics, the maze was thoroughly cleaned between trials with diluted ethanol.

### Western blot

Immediately after finishing the H-W mazes, mice were euthanized (100 μl i.p. injection of euthasol; Sigma Aldrich, Oakville, ON, Canada), their brains removed and tissue blocks were cut using a stainless steel brain matrix (1 × 1.5 × 0.75 inches). Both dorsal hippocampi were dissected according to a mouse atlas and frozen on dry ice (Paxinos and Franklin, 2001). Western blots were then prepared as described previously (Choeiri et al., 2006). Briefly, hippocampi were homogenized over ice in a homogenate buffer/protease inhibitor cocktail (Sigma Aldrich, Oakville, ON, Canada). The homogenates were centrifuged, protein content was quantified using a standard BSA kit (Pierce, Rockford, IL, USA) and samples were frozen at −80°C until further analysis. Proteins were loaded at a concentration of 300 μg/ml and samples in quadruplicate (12 μg/lane) were resolved by SDS-PAGE. Proteins were then transferred to pure nitrocellulose membranes and blocked for 1 h in 5% skim milk and 10 M phosphate buffered saline (PBS) solution at room temperature. Antibody specificity was determined prior to commencing Western blot analyses on experimental animals by confirming a single band of binding of the protein of interest at the appropriate molecular weight. Optimal concentrations of primary/secondary antibody were then confirmed by serial dilutions. Membranes were incubated in 5% skim milk and TBST (20 mM Tris/HCl, 137 mM NaCl, 0.4% Tween 20, pH 7.6) solution with monoclonal anti-PSD-95 antibody (1:2000; Millipore Corporation, Burlington, ON, Canada) and monoclonal anti-β-tubulin antibody (1:10,000; Sigma Aldrich, Oakville, ON, Canada) at 4°C overnight. After 3 × 10 min washes in TBST, fluorescent Alexa 680-linked antibody (1:10,000, Molecular Probes, Burlington, ON, Canada) and IR 800 antibody (1:10,000; LI-COR Biosciences, Lincoln, NE, USA) in 5% skim milk and TBST solution were applied for 1 h at 4°C. After 3 × 10 min washes in TBST, Western blots were scanned using the Odyssey infra-red system (LI-COR Biosciences, Lincoln, NE, USA) in 700 and 800 nm channels in a single scan at 169 μm resolution. Simultaneous detection of two fluorescent antibodies (i.e., Alexa 680 and IR 800) allowed for the measurement of PSD-95 and β-tubulin proteins within each sample. The density of each protein band of interest was measured, background subtracted and normalized to β-tubulin by the LI-COR analysis software.

### Statistical analyses

Latency to complete the H-W mazes, number of errors, as well as hippocampal PSD-95 levels in MPEP treated mice compared with saline treated controls were the variables of interest in this study. Using SPSS 19 (IBM Canada Ltd., Markham, Canada), latency was analyzed by a 2 × 7 × 5 mixed-design ANOVA with treatment (MPEP; saline) as the between-subjects variable and both maze (seven levels) and trial (five levels) as the repeated measures variables. Similarly, the number of errors made on the H-W mazes was analyzed by a separate 2 × 7 × 5 mixed-design ANOVA. Prior to analyses, data were evaluated to ensure that assumptions underlying mixed-design ANOVA were met. These preliminary analyses indicated that the majority of the latency as well as error data were skewed, and consequently, these variables were subjected to log_10_ transformations in order to normalize the distributions of the data. Following log_10_ transformation, neither latency nor error data were identified as outliers (>four SDs from the group mean; Van Selst and Jolicoeur, 1994). There were no missing data in this study.

In order to confirm the effectiveness of MPEP treatment, each mouse underwent the marble burying assay immediately after each test maze. These data remained skewed following square root, log_10_, and inverse transformations and therefore were not amenable to a 2 × 7 ANOVA analysis. As such, data were analyzed using several non-parametric two independent sample, Mann-Whitney *U*-tests. Specifically, analyses focused on the number of marbles buried following completion of each maze as a function of treatment (MPEP; saline). Assumptions underlying the Mann-Whitney *U*-tests were met prior to running the analyses.

To examine protein levels following mGluR-5 antagonist treatment, an independent samples *t*-test was performed with treatment (MPEP; saline) as the independent variable and the protein ratio of PSD-95 normalized to a control protein, β-tubulin, as the dependent variable. To ensure equal loading of protein samples across groups, an additional *t*-test was conducted with treatment as the independent variable (MPEP; saline) and β-tubulin as the dependent variable. Prior to analyses, data were evaluated to ensure that assumptions underlying independent samples *t*-test were met.

In order to examine the association between protein expression and behavioral performance, three separate bivariate correlations (Pearson’s *r*) were conducted. The correlational analyses were based on relative PSD-95 protein levels (normalized to β-tubulin) and mean total errors on the H-W mazes, defined as aggregate errors divided by the total number of learning trials (maze × trials = 35). Specific correlations focused on the relationship between PSD-95 protein levels and mean errors from: (1) *Fmr1* KO maze runners of both treatments; (2) MPEP treated runners only; and (3) saline treated runners only. As a control, correlations were also performed between β-tubulin protein levels and mean total errors from #1.

Given the *a* *priori* hypotheses that specified the direction of the effect in each of the aforementioned correlations, one-tailed tests of significance for the correlational coefficients were conducted.

## Results

A 2 × 7 × 5 mixed measures ANOVA was conducted to evaluate the effects of treatment (MPEP; saline) as the between-groups measure and repeated measures of both maze (seven levels) and trial (five levels) on the latency to complete the H-W mazes. There was a main effect for maze, *F*_(5, 94)_ = 3.01, *p* = 0.01, partial *η*^2^ = 0.13, and for trial, *F*_(3, 67)_ = 60.12, *p* < 0.001, partial *η*^2^ = 0.75, but not for treatment, *F*_(1, 20)_ = 1.45, *p* = 0.24, partial *η*^2^ = 0.07, indicating that the latency to complete the mazes did not differ between MPEP and saline treated mice. There was also a significant interaction between treatment and maze, *F*_(5, 94)_ = 4.06, *p* = 0.003, partial *η*^2^ = 0.17, as well as maze and trial *F*_(9, 189)_ = 1.89, *p* = 0.05, partial *η*^2^ = 0.08. However, the interaction between treatment and trial *F*_(3, 67)_ = 1.07, *p* = 0.37, partial *η*^2^ = 0.05 was not significant. Likewise, the three-way interaction between treatment, maze, and trial was not significant *F*_(9, 189)_ = 1.01, *p* = 0.44, partial *η*^2^ = 0.05.

**Figure 2:**
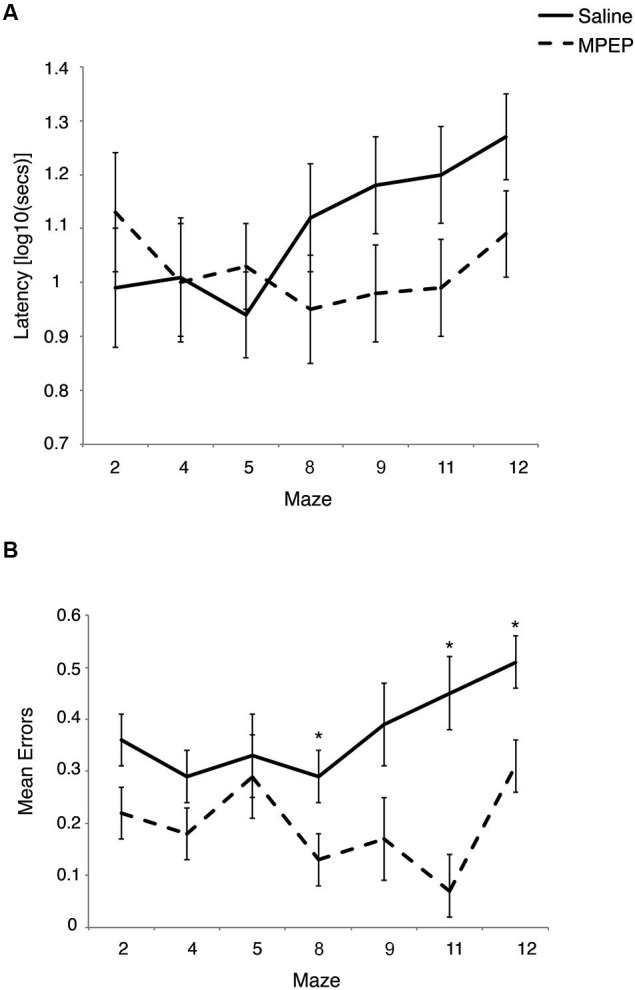
**(A)** Latency to complete each Hebb-Williams (H-W) test maze for *Fmr1* KO mice treated with saline or MPEP. Drug treatment did not statistically affect completion times between groups. Error bars represent the S.E.M. **(B)** Mean errors collapsed across trials for each H-W test maze for *Fmr1* KO mice treated with saline or MPEP. Mice treated with MPEP made significantly fewer errors on mazes #8, 11 and 12. Error bars represent the S.E.M; * *p* < 0.007.

Bonferroni corrections were made to the *α*-level of 0.05 before exploring simple main effect analyses of treatment within maze, resulting in *p* < 0.007 (0.05/7 = 0.007) for significance. These analyses indicated that there were differences in the latencies between MPEP and saline treated mice on maze #9, *F*_(1, 20)_ = 5.08, *p* = 0.04, partial *η*^2^ =0.20, maze #11, *F*_(1, 20)_ = 5.36, *p* = 0.03, partial *η*^2^ = 0.21 and maze #12, *F*_(1, 20)_ = 6.08, *p* = 0.02, partial *η*^2^ = 0.23. However, given the adjustment to guard against Type I error, these differences were deemed not statistically significant. Given the similar latencies to complete maze running between drug and vehicle groups, these findings are consistent with previous research indicating that MPEP treatment does not significantly reduce locomotor activity (Figure 2A).

Bonferroni corrections were made to the *α*-level of 0.05 before exploring simple main effect analyses of trial within maze, resulting in *p* < 0.007 (0.05/7 = 0.007) for significance. These analyses indicated that there were differences in the latencies between trials on maze #2, *F*_(4, 17)_ = 7.64, *p* = 0.001, partial *η*^2^ =0.64, maze #4, *F*_(4, 17)_ = 23.36, *p* = 0.000001, partial *η*^2^ = 0.85, maze #5, *F*_(4, 17)_ = 6.22, *p* = 0.003, partial *η*^2^ = 0.59, maze #8, *F*_(4, 17)_ = 11.05, *p* = 0.0001, partial *η*^2^ =0.72, maze #9, *F*_(4, 17)_ = 11.26, *p* = 0.0001, partial *η*^2^ = 0.73, maze #11, *F*_(4, 17)_ = 12.54, *p* = 0.0001, partial *η*^2^ = 0.75, but not on maze #12, *F*_(4, 17)_ = 4.68, *p* = 0.01, partial *η*^2^ = 0.52.

Pairwise comparisons on the latency data adjusted to control for the effects of comparing mean trial differences within each maze (*α* = 0.05/60 = 0.0008) showed that on maze #2, *Fmr1* KO mice were significantly slower on trial 1 relative to completion times on trials 3 and 4. In addition they were significantly slower on trial 2 compared to trial 4. On maze #4, mice were slower on trial 1 compared to their completion times on trials 3, 4 and 5; whereas trial 2 took longer to complete than trial 4. On maze #5, mice took longer to complete trial 1 compared with run times on trials 4 and 5. Subsequently, mice completed maze #8 slower on trial 1 compared to trials 2, 3, 4 and 5, whereas trial 4 was completed faster than trial 2. During maze #9, latencies were again slower on trial 1 compared with trials 3, 4 and 5. Finally on maze #11, run times were quicker on trials 2, 3 and 5 relative to trial 1. Thus, despite some variability in the trial by maze interaction data, pairwise comparisons indicated latencies were longest for trial 1 and in general, tended to decrease with increased repetition, as would be expected if mice were learning the maze configuration and motivated to obtain the food reward.

Regarding error data, a 2 × 7 × 5 mixed measures ANOVA was conducted to evaluate the effects of treatment (MPEP; saline) as the between-groups measure and repeated measures of both maze (seven levels) and trial (five levels) on the number of errors committed on the H-W mazes. There was a main effect for treatment *F*_(1, 20)_ = 63.71, *p* < 0.001, partial *η*^2^ =0.76, for maze, *F*_(4, 84)_ = 4.13, *p* = 0.004, partial *η*^2^ = 0.17, indicating that the number of errors made on the mazes differed between MPEP and saline treated mice. There was also a main effect for trial, *F*_(3, 65)_ = 24.43, *p* < 0.001, partial *η*^2^ = 0.55. There was a significant interaction between treatment and maze, *F*_(4, 84)_ = 2.82, *p* = 0.03, partial *η*^2^ = 0.12, whereas the interaction between treatment and trial *F*_(3, 65)_ = 2.22, *p* = 0.09, partial *η*^2^ = 0.10 approached significance. However, the interaction between maze and trial *F*_(9, 188)_ = 1.32, *p* = 0.23, partial *η*^2^ = 0.06 was not significant. Likewise, the three-way interaction between treatment, maze, and trial was not significant *F*_(9, 188)_ = 0.94, *p* = 0.50, partial *η*^2^ = 0.04.

Bonferroni corrections were made to the *α*-level of 0.05 before exploring simple main effect analyses of treatment within maze, resulting in *p* < 0.007 (0.05/7 = 0.007) for significance. These analyses indicated that there were significantly less errors committed by MPEP treated mice on maze #2, *F*_(1, 20)_ = 6.21, *p* = 0.02, partial *η*^2^ = 0.24, maze #4, *F*_(1, 20)_ = 5.94, *p* = 0.02, partial *η*^2^ = 0.23, maze #8, *F*_(1, 20)_ = 8.67, *p* = 0.007, partial *η*^2^ = 0.30, maze #9, *F*_(1, 20)_ = 7.53, *p* = 0.01, partial *η*^2^ = 0.27, maze #11, *F*_(1, 20)_ = 30.80,* p* = 0.00002, partial *η*^2^ = 0.61, and maze #12, *F*_(1, 20)_ = 17.28,* p* = 0.0005, partial *η*^2^ = 0.46, However, when adjustments were made to guard against Type I error, MPEP treated mice committed significantly fewer errors on only three mazes relative to saline controls (mazes #8, 11, 12). Combined, these data indicate that on several of the H-W mazes, MPEP administration results in significantly less errors than in *Fmr1* KO mice treated with saline only (Figure 2B).

**Figure 3:**
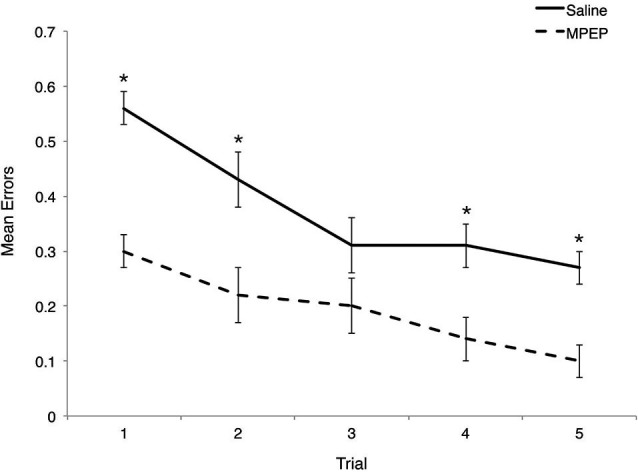
**Mean errors on Hebb-Williams (H-W) test mazes for *Fmr1* KO mice treated with saline or MPEP across trials.** MPEP treated *Fmr1* KO mice made significantly fewer errors on trials 1, 2, 4, and 5. Error bars represent the S.E.M; * *p* < 0.005.

Bonferroni corrections were made to the *α*-level of 0.05 before exploring simple main effect analyses of treatment within trial, resulting in *p* < 0.01 (0.05/5 = 0.01) for significance. MPEP administration resulted in significantly fewer errors on trial 1, *F*_(1, 20)_ = 54.95, *p* = 0.0000004, partial *η*^2^ = 0.73, trial 2, *F*_(1, 20)_ = 17.16, *p* = 0.001, partial *η*^2^ = 0.46, trial 4, *F*_(1, 20)_ = 21.62, *p* = 0.0002, partial *η*^2^ = 0.52, and trial 5, *F*_(1, 20)_ = 25.97, *p* = 0.0001, partial *η*^2^ = 0.57. Unexpectedly, treatment had no effect on the mean errors observed on trial 3, *F*_(1, 20)_ = 4.43,* p* = 0.05, partial *η*^2^ = 0.18. Thus, MPEP treatment reduces errors committed on four out of five trials with the biggest impact (as reflected by effect size) occurring on the first trial (Figure 3).

**Figure 4:**
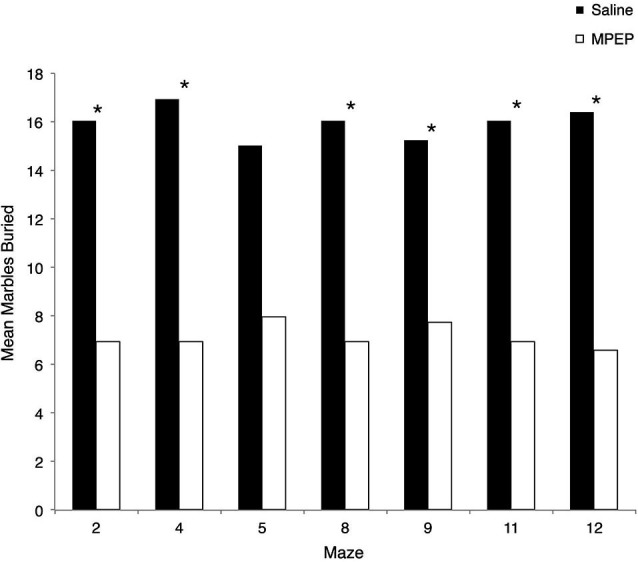
**Mean marbles buried across each Hebb-Williams (H-W) test maze for *Fmr1* KO mice treated with saline or MPEP.** MPEP treated mice buried significantly less marbles following each maze except for maze 5. Data were analyzed by non-parametric, Bonferroni corrected Mann Whitney *U*-tests; * *p* < 0.007.

Several Mann-Whitney *U*-tests were conducted to evaluate whether MPEP remained at physiologically active levels during the experiments. Bonferroni corrections were made to the *α*-level of 0.05 before performing these tests, resulting in *p* < 0.007 (0.05/7 = 0.007) for significance. The results of the Mann-Whitney *U*-tests were in the expected direction and significant such that MPEP treated mice were found to bury significantly more marbles than saline treated controls for maze #2, *z* = −3.306, *p* = 0.001 (MPEP average rank = 6.95; Saline = 16.95), maze #4, *z* = −3.31, *p* = 0.001 (MPEP average rank = 6.95; Saline = 16.05), maze #8, *z* = −3.30, *p* = 0.001 (MPEP average rank = 6.95; Saline = 16.05), maze #9, *z* = −2.74, *p* = 0.006 (MPEP average rank = 7.73; Saline = 15.27), maze #11, *z* = −3.34, *p* = 0.001 (MPEP average rank = 6.95; Saline = 16.05), and maze #12, *z* = −3.56, *p* < 0.001 (MPEP average rank = 6.59; Saline = 16.41). With the adjusted level of *α*, the number of marbles buried was not statistically different between MPEP and saline treated mice for maze #5, *z* = −2.58, *p* = 0.01 (MPEP average rank = 7.95; Saline = 15.05). Taken together, the marble burying assay following the completion of each of the H-W test mazes confirmed that the MPEP treatment was physiologically active during the test phases (Figure 4).

**Figure 5:**
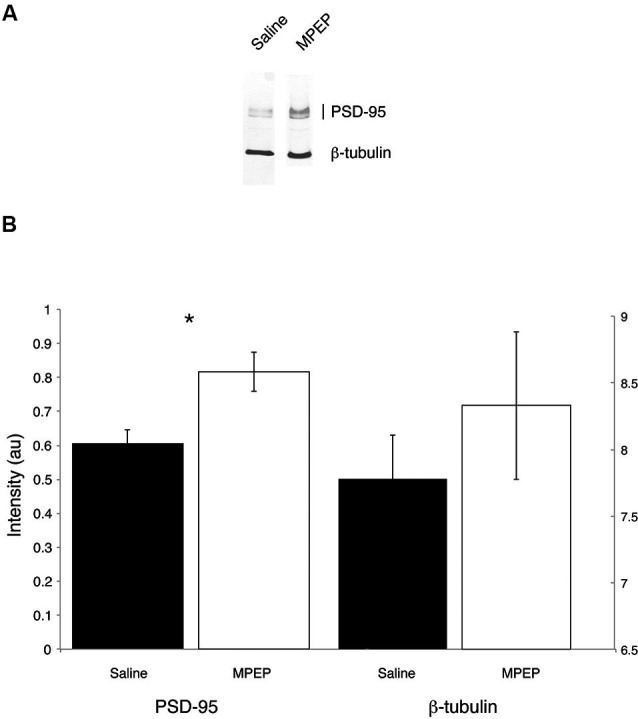
**Representative Western blots from dorsal hippocampi of *Fmr1* KO mice treated with saline or MPEP for protein expression of PSD-95 and β-tubulin.** PSD-95 is found around the expected molecular weight of 95 kDa and β-tubulin is found at 55 kDa. PSD-95 levels are rescued in MPEP treated *Fmr1* KO mice only. Error bars represent the S.E.M; * *p* < 0.025.

Independent sample *t*-tests were performed to evaluate the hypothesis that mGluR-5 antagonist treatment could selectively rescue hippocampal PSD-95 protein levels. Hippocampal β-tubulin levels were also measured because this housekeeping protein was not expected to vary with treatment condition. Bonferroni corrections were made to the *α*-level of 0.05 before performing these tests, resulting in *p* < 0.025 (*α* = 0.05/2 = 0.025) for significance. The *t*-tests indicated that PSD-95 levels were significantly higher in MPEP treated mice compared with vehicle condition, *t*_(20)_ = 3.00, *p* = 0.007, 95% CI [0.064, 3.56], whereas there were no differences in β-tubulin levels between MPEP and vehicle treated mice, *t*_(20)_ = 0.851, *p* = 0.40, 95% CI [−0.80, 1.89]. The effect size as reflected by, *η*^2^, indicated that 31% of the variance in PSD-95 levels was accounted for by whether or not mice received MPEP/vehicle treatment whereas only 0.03% of the variance in β-tubulin levels was accounted for the treatment. These data suggest that mGluR-5 antagonism has an augmenting affect on the levels of the scaffolding protein PSD-95 (Figure 5).

**Figure 6:**
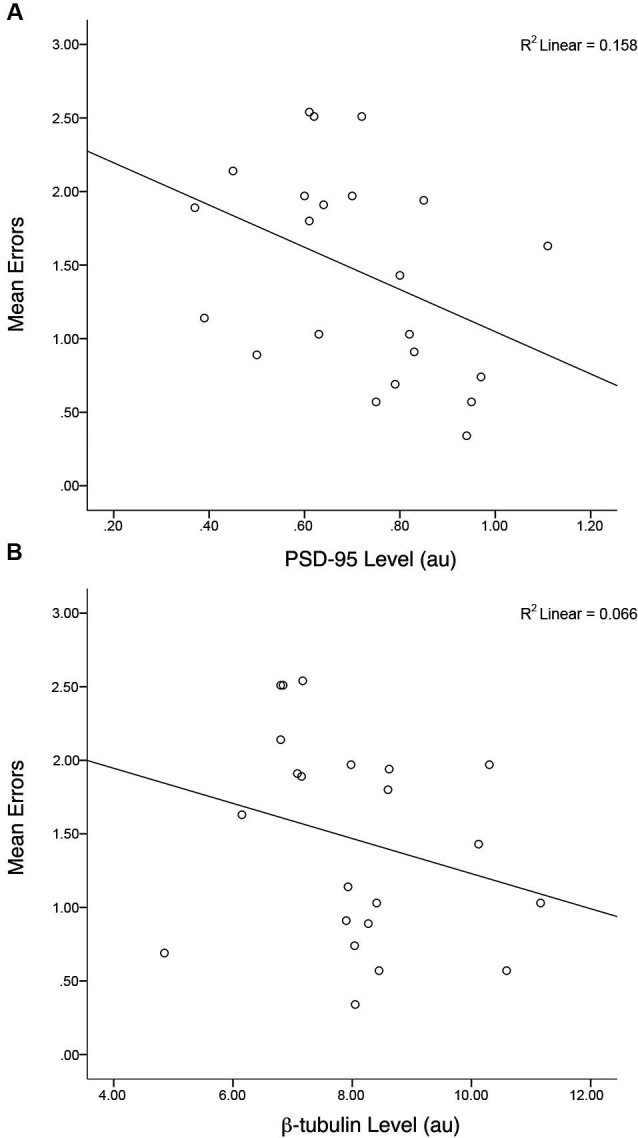
**Levels of hippocampal PSD-95 but not control protein (β-tubulin) are correlated with behavioral performance.** A negative correlation between mean errors on the Hebb-Williams (H-W) mazes and PSD-95 protein levels was observed, *r*_(20)_ = −0.40, *p* = 0.03, *r*^2^ = 0.16. * *p* < 0.05.

Similar to the group data from Gandhi et al. (2014; that comprised the entire sample of animals) a correlation of all *Fmr1* KO mice, irrespective of treatment, indicated there was a negative association between PSD-95 levels and mean total errors on the H-W mazes, *r*_(20)_ = −0.40, *p* = 0.03, *r*^2^ = 0.16 (Figure 6). This association was not evident when examining the correlation between β-tubulin levels and mean total errors from *Fmr1* KO mice, *r*_(20)_ = −0.26, *p* = 0.12, *r*^2^ = 0.06. Within treatment groups, there were no relationships between the PSD-95 levels of MPEP treated mice and mean total errors,* r*_(9)_ = −0.042, *p* = 0.45, *r*^2^ = 0.01, nor saline treated mice and mean total errors, *r*_(9)_ = 0.28, *p* = 0.21, *r*^2^ = 0.08. In addition, after adjusting the *α*-levels to control for repeated tests, (0.05/4 = 0.012) only the initial correlation consisting of the entire sample of *Fmr1* KO mice trended towards significance. Collectively, these data confirm that as PSD-95 levels increase, mean errors on the H-W mazes decrease and *vice versa*.

## Discussion

FXS is a debilitating mental, physical, and behavioral condition that occurs due to lack of expression of the Fragile X Mental Retardation 1 protein (FMRP; reviewed in Santoro et al., 2012). The altered expression results in a number of characteristic symptoms including disorders of intellectual development and frequently co-morbid autism spectrum disorder. Visual-spatial impairment is part of the cognitive profile in FXS and was the focus of the present investigation. Despite a common finding in the research literature that hippocampal lesions impair performance on tasks of spatial navigation and learning (Morris et al., 1982; Sutherland et al., 1982; Jarrard, 1993; Hock and Bunsey, 1998; Lee and Kesner, 2003; Clark et al., 2005; Okada and Okaichi, 2009), inconsistent results have been reported when testing *Fmr1* KO mice. These differences may be a function of variability in the background strain used or the assays employed. In the present study we employed maze learning tasks, the H-W mazes, previously shown to be sensitive to detecting dorsal hippocampal deficits (Shore et al., 2001; Rogers and Kesner, 2006) including in a murine model of FXS, *Fmr1* KO mice. Concomitant with greater errors committed by *Fmr1* KO as compared to wild type mice (MacLeod et al., 2010), PSD-95, a hippocampal protein involved in synaptic plasticity and a target of Fmrp, is selectively upregulated in wild type but not KO mice (Gandhi et al., 2014). We demonstrate here that a selective antagonist for mGluR-5, MPEP, reverses both behavioral deficits in *Fmr1* KO mice, as evidenced by fewer errors in treated vs. saline treated animals on most H-W maze problems, as well as the molecular deficit of interest, that is, PSD-95 levels. These results provide support for the importance of mGluR-5 signaling generally, and PSD-95, in particular, in the pathophysiology of FXS and autism spectrum disorder.

Although the molecular mechanisms of synapse modifications at dendritic spines are unknown, one perspective is that certain scaffolding proteins maintain the long-term transmission efficiency of a synapse (Ehrlich and Malinow, 2004; McCormack et al., 2006). In this scenario, scaffolding proteins are thought to serve as placeholders or slot proteins for receptors such as AMPARs. PSD-95 has been proposed to possess many qualities of a slot protein (Schnell et al., 2002) because it is more stable than other post-synaptic density (PSD) proteins such as CaMKII*α*, CaMKIIβ, GluR2 or Stargazin, consistent with a role in regulating the PSD (Sturgill et al., 2009). Levels of PSD-95 were reported to be redistributed to dendrites in the visual cortex following eye opening in litters of rodents, and these changes lasted upwards of 6 h and were contingent on sustained environmental experience (Yoshii et al., 2003). Moreover, changes in the sizes of individual PSDs over days were associated with changes in PSD-95 retention times and PSD-95 increased with developmental age and dropped sharply following sensory deprivation (Gray et al., 2006). Importantly, in FXS, there is evidence that PSD-95 is dysregulated. Specifically, increased translational levels were observed during basal states in *Fmr1* KO as compared to wild-type mice as well as relatively low protein levels following stimulus induction in this genotype. PSD-95 mRNA transcripts were also found to selectively deteriorate in the hippocampus but not in the cortex or cerebellum of *Fmr1* KO mice (Todd et al., 2003; Muddashetty et al., 2007; Zhu et al., 2011).

Pharmacological treatments blocking mGluR-5 receptors can stabilize basal protein translation levels and this approach has been hypothesized as a means of ameliorating some of the core symptoms of FXS, including disorders in intellectual development (Dölen and Bear, 2005; Bhakar et al., 2012). In studies using drosophila KO (*dfmr1*) and *Fmr1* KO murine models, the use of mGluR-5 antagonists has been successful in correcting many features of FXS including elevated and inappropriately expressed protein levels at basal states, decreasing frequency of audiogenic seizures, reversing excessive AMPA internalization, reducing the number of abnormally thin dendritic spines, and reversing behavioral/learning deficits (McBride et al., 2005; Yan et al., 2005; Nakamoto et al., 2007; de Vrij et al., 2008; Pan et al., 2008; Choi et al., 2010; Osterweil et al., 2010; Levenga et al., 2011; Su et al., 2011; Tauber et al., 2011). Despite the rescue of many phenotypic features of FXS, the identification of the specific proteins underlying these functions remains to be elucidated. Theoretically, the stabilization of PSD-95 protein in *Fmr1* KO mice would allow for improved local regulation during periods of synaptic plasticity while learning the H-W mazes.

Our Western blot analyses following completion of the H-W mazes revealed that MPEP treated mice had statistically higher PSD-95 protein levels. This effect was specific to PSD-95 since levels of the control protein (β-tubulin) remained unchanged across treatment conditions. Thus, our findings suggests that PSD-95 protein deficits can be rescued by targeting mGluR-5 receptors. An additional implication pertains to the broader question of “when” it is appropriate to intervene with pharmacological treatment. As FXS is a developmental disorder, the vast majority of animal model studies have targeted intervention at the embryonic stages or very early in post-natal life. Conceptually, it is of great interest to determine if the FXS phenotype can be corrected after symptom onset. If not, it would suggest that a critical therapeutic window has been missed and argue against the idea that the symptoms of FXS are caused by ongoing irregularities of synaptic signaling (Michalon et al., 2012). This question was addressed in a study examining *Fmr1* KO mice aged 4–5 weeks with anatomically developed and highly plastic brains, corresponding to young adults. Specifically, treatment with an mGluR-5 inhibitor corrected learning and memory deficits in an inhibitory avoidance paradigm, improved dendritic spine abnormalities, and ameliorated elevated Extracellular signal-regulated kinase (ERK) and mTOR kinase activation (pathways previously shown to underlie the pathophysiology of FXS). Our data, which suggest reversal of molecular and behavioral deficits, are consistent with these findings (Michalon et al., 2012). In addition, since the* Fmr1* KO mice in our study were 12 weeks or older before beginning behavioral testing, our findings further demonstrate that a model of the FXS phenotype can be corrected in aged mice roughly corresponding to adulthood.

The behavioral data from the H-W mazes were analyzed according to two dependent variables of interest, latency and error. Regarding the former, analyses of the treatment by maze interaction indicated that there were differences in the latency between MPEP and saline treated mice on several mazes. Owing to high levels of variability in the runs times, faster completion times by MPEP treated mice were not statistically different from controls. However, the similar latency to complete mazes between drug and vehicle groups indicates that our data are consistent with previous research demonstrating that MPEP treatment does not adversely affect locomotor activity (Yan et al., 2005; Silverman et al., 2010; Mehta et al., 2011; Thomas et al., 2012). Collapsed across treatment, we also observed that latency of maze completion was longest for trial 1 and generalizing across mazes, tended to decrease with increased repetition. Thus, both groups of mice were capable of improving their latency performance with increased exposure to the mazes.

Consistent with our hypothesis, on mazes deemed more challenging (#8, 9, 11, 12; Shore et al., 2001), MPEP treated mice made significantly fewer errors (i.e., #8, 11, 12). When examining the behavioral performance of the mazes deemed more difficult, on maze #8, saline treated mice continued to explore previously unsuccessful routes towards the goal box whereas MPEP treated mice demonstrated a reduction in errors over trials. Counterintuitively, there were no differences between drug and saline treated mice on maze #9, which may reflect the variability in the data set or a lack of difficulty of this maze for this background strain. Qualitatively, on mazes #11 and #12, saline treated controls committed more perseverative errors, circling isolated and removed barriers from the goal box, thereby getting stuck in unsuccessful “loops”. That MPEP treated mice did not commit such responses suggests that MPEP treatment may correct perseveration, a common cognitive feature of FXS (Hooper et al., 2008).

Finally, the treatment by trial interaction data revealed that MPEP treated mice made significantly fewer errors on trials #1, 2, 4, and 5 relative to controls. Given that the largest effect size occurred on the first trial, this suggests that MPEP may also have corrected impulsive responding, which is another feature that is commonly observed in FXS (Hagerman, 2002).

The pharmacological efficacy of MPEP was confirmed with a marble burying assay immediately following the test phase in order to validate our findings. Marble burying, a repetitive behavior, has been shown to be decreased following the administration of Grp I mGluR antagonists (Spooren et al., 2000; Thomas et al., 2012). In the present investigation, MPEP treated mice buried significantly fewer marbles than controls after the completion of all mazes (thus confirming drug efficacy), with the exception of #5. It is unclear why fewer marbles relative to controls were buried here, however as there were no error differences between treatment groups for this maze; interpretation of our findings is not affected by this result.

Overall, our correlational findings are inconclusive and merit further investigation. Although we replicated a negative correlation between PSD-95 levels and mean errors for the entire sample of mice, as found in our previous study (Gandhi et al., 2014), we did not demonstrate a statistically significant relationship within the treatment groups. We suspect that larger sample sizes of mice will provide the necessary power to allow us to characterize this relationship appropriately.

Whether pharmacological studies of mGluR-5 antagonists in mouse models of FXS will translate into effective treatments for human patients remains to be determined. To date, only two studies have been completed in patients affected by FXS. A pilot study was conducted to determine pharmacokinetics and side effects of a single dose trial of the mGluR-5 antagonist, fenobam, to 12 male and female FXS patients (Berry-Kravis et al., 2009). Pre/post outcome measures included prepulse inhibition (PPI) and the continuous performance test (CPT) to assess sensory gating, attention and inhibition. The results indicated there were no adverse reactions to the fenobam administration and PPI improved by at least 20% in half of the sample relative to baseline. By comparison, performance on the CPT did not improve although this finding was attributable to ceiling effects. The other study employing an mGluR-5 antagonist was conducted using AFQ056 in 30 male FXS patients ranging in age from 18–35 (Jacquemont et al., 2011). These researchers initially did not find any improvement in behavioral symptoms of FXS following treatment as assessed by the Aberrant Behavior Checklist-Community Edition (ABC-C). However, a subset of the patients who had the full *Fmr1* promoter methylation and no detectable *Fmr1* mRNA improved significantly more on the ABC-C and the Repetitive Behavior Scale following treatment compared with placebo. Since those patients with partial promoter methylation did not show behavioral improvement following AFQ056 treatment, the authors posited that mGluR-5 antagonism might be better suited for FXS patients with full methylation at the *Fmr1* promoter. mGluR-5 antagonists are not the only receptor mechanism/molecular target under investigation. FXS is a complex neurodevelopmental disorder and Fmrp regulates signaling by other receptors as well. Therefore, antagonism of Group I mGluR signaling is not likely to produce beneficial therapeutic effects for every patient. Moreover, there are other aspects of the FXS phenotype that are unrelated to mGluR function. Other research has focused on other targets and agents such as GABA-A and B receptors, ampakines, brain derived neurotrophic factor (BDNF), aripiprazole, lithium and intracellular signaling pathways via phosphatase and kinase inhibitors. In all likelihood, patients will display varied outcomes to different targeted treatments based on interplay between genetics, intracellular neuronal pathways, and synaptic function (Gross et al., 2012).

FXS is the most common single gene disorder associated with autism spectrum disorder (Hagerman et al., 2010) and there are numerous commonalities between FXS and autistic spectrum disorder. Similar to FXS, many autistic patients suffer from seizure disorder and cognitive impairment (Canitano, 2007). There is also delayed language acquisition and repetitive behaviors (Hagerman, 1996) and 25–47% of FXS patients have a diagnosis of autism (Kaufmann et al., 2004; Hatton et al., 2006). Models of FXS are potentially advantageous to autism because Fmrp controls the translation of plasticity proteins implicated in autism such as neuroligins and SHANK proteins (Darnell et al., 2011). Moreover, low levels of FMRP relative to controls have been reported in autism spectrum disorder (Fatemi and Folsom, 2011). Using a Black and Tan BRachyury TBR T+ Itpr3tf/J (BTBR) murine model of autism, one study reported that MPEP treatment ameliorated repetitive self-grooming behavior without significant sedating side effects (Silverman et al., 2010). Likewise, in a valproic acid (VPA) murine model of autism, MPEP reduced excessive self-grooming as well as marble burying behavior (Mehta et al., 2011). Although further study is needed, preliminarily, MPEP appears to be a suitable pharmacological intervention for both FXS and autistic disorder. Our findings indicate that MPEP treatment can reverse PSD-95 protein deficits and errors on more complicated H-W test mazes. Given the significant phenotypic overlap between FXS and autism as well as the lack of a viable behavioral assay to test symptoms improvement in the autism field, the use of the H-W mazes with murine models of autism spectrum disorder appears promising and warrants further investigation.

## Conflict of interest statement

The authors declare that the research was conducted in the absence of any commercial or financial relationships that could be construed as a potential conflict of interest.
